# Ultra-Processed Food Intakes and Health Outcomes in Adults Older Than 60 Years: A Systematic Review

**DOI:** 10.1093/nutrit/nuae223

**Published:** 2025-01-31

**Authors:** Fay A Shahatah, Tom R Hill, Andrea Fairley, Anthony W Watson

**Affiliations:** Faculty of Medical Sciences, Population Health Sciences Institute, Newcastle University, Newcastle upon Tyne NE2 4HH, United Kingdom; Faculty of Medical Sciences, Population Health Sciences Institute, Newcastle University, Newcastle upon Tyne NE2 4HH, United Kingdom; Faculty of Medical Sciences, Population Health Sciences Institute, Newcastle University, Newcastle upon Tyne NE2 4HH, United Kingdom; Faculty of Medical Sciences, School of Biomedical, Nutritional and Sports Sciences, Newcastle University, Newcastle upon Tyne NE2 4HH, United Kingdom; Faculty of Medical Sciences, Population Health Sciences Institute, Newcastle University, Newcastle upon Tyne NE2 4HH, United Kingdom; Faculty of Medical Sciences, School of Biomedical, Nutritional and Sports Sciences, Newcastle University, Newcastle upon Tyne NE2 4HH, United Kingdom

**Keywords:** ultra-processed food, food processing, Nova food classification, older adults, observational study

## Abstract

**Context:**

Relationships between ultraprocessed food (UPF) intakes and health have been well described in adults, but evidence of these relationships in older adults is rather limited. Demographic shifts in aging emphasize the importance of understanding the role of diet in maintaining health during later life.

**Objective:**

The aim of this systematic review was to identify health outcomes associated with UPF consumption in those aged ≥60 years.

**Data Sources:**

The Medline, Web of Science, Scopus, and Embase databases were searched from inception until June 2023, using search terms representing UPF and any health outcome. Observational studies restricted to individuals aged ≥60 years using the Nova food classification were included. Articles were screened independently by 2 researchers using PECOS inclusion criteria.

**Data Extraction:**

Two-thirds of the studies in the present review were prospective cohort studies (n = 4) and the rest were cross-sectional (n = 2). The number of participants ranged from 652 to 2713 across the 6 studies. The mean age of participants ranged between 67 and 73 years.

**Data Analysis:**

In the prospective cohort studies, the highest consumption of UPF was associated with incident frailty, incident dyslipidemia, renal function decline, and abdominal obesity. For cross-sectional studies, the highest intake of UPF was associated with negative outcomes similar to young and middle-aged cohorts, with the addition of nutritional frailty and worse cognitive performance.

**Conclusions:**

Currently, the limited understanding of how high UPF intakes could be associated with negative health outcomes for older adults influences the ability to inform policy and practice recommendations. More research is required owing to the very limited number of studies, specifically in older adults.

**Systematic Review Registration:**

PROSPERO registration no. CRD42023442783.

## INTRODUCTION

In recent decades, the number of older adults (aged ≥60 years) has increased globally by 162%, from 382 million in 1980 to 1 billion in 2019.^1^ Projections suggest this trend will persist, with the number of older adults anticipated to nearly double, reaching approximately 2.1 billion by 2050.^1^ As people attain longer lifespans, novel health challenges emerge. These can be characterized by a higher prevalence of geriatric syndromes such as frailty and disability, as well as other common health conditions that result from a mix of factors not classified as diseases of specific organs,^2^ or comorbid conditions, such as malnutrition and sarcopenia. Sarcopenia is an age-related disease characterized by muscle loss and loss of physical function. A recent systematic review and meta-analyses indicated that older adults with sarcopenia consume significantly less protein than their counterparts without sarcopenia.^3^^,^^4^ Furthermore, the aging process entails a spectrum of age-related change encompassing physical impairment, physiological alterations, chronic diseases, as well as psychological and psychosocial changes,^5^ all of which can significantly influence food choices and nutritional status.^6^^,^^7^ Extensive research on older adults has been conducted that underscores the importance of various aspects of a healthy diet (including whole foods, dietary patterns, and nutrients) to overall health and the promotion of healthy aging.^8–12^ For this reason, a position statement was produced by the UK Scientific Advisory Committee on Nutrition acknowledging limitations within the current literature and recommending the need for research to explicitly consider nutrition and health in adults aged ≥65 years.^13^

Low intakes of fiber, protein, vitamins, and minerals are well-documented characteristics of the diets of adults consuming high amounts of ultraprocessed foods (UPFs). These industrially processed foods contain limited whole foods and an abundance of food additives, as defined by the Nova food classification.^14^ UPF encompasses a wide range of food items, including breakfast cereals, packaged snacks, processed breads, flavored yogurt, and frozen meals.^5^ In a British longitudinal cohort study of 85-year-old participants, many foods classified as UPFs, including cereals and cereal products (CCP; which include bread and breakfast cereals), were consumed by at least 75% of the cohort, making CCP the top contributors of overall energy and fiber intake.^15–22^ Furthermore, the highest intake of folate, iron, and selenium were identified in CCP, with contributions of 31.5%, 49.2%, and 46.7%, respectively. These findings suggest that fortified foods (which can be classed as UPFs) are an important source of some micronutrients among older adults.^23^

Over the past decade, the consumption of UPFs has received particular attention with regard to links to their high consumption by and poor health outcomes in adults.^15–22^ Previous meta-analyses and epidemiological studies, encompassing >10 million participants, have extensively examined the relationship between UPF consumption and a wide spectrum of adverse health outcomes and death.^24^^,^^25^ It is important to note that the nature of this research is predominantly observational, relying on secondary analyses of cohort and cross-sectional studies. These investigations typically involve comparisons between groups with varying levels of UPF consumption within populations and their respective associations with health outcomes. Although these findings offer valuable insight into the association between UPFs and health, it is important to acknowledge the inherent limitations of such studies. These limitations include a scarcity of direct evidence establishing a causal relationship between UPF intake and adverse health outcomes, as well as the inclusion of diverse age groups within study cohorts.

Additionally, many studies used statistical adjustments for age in their analyses to mitigate the potential skewing of results by age-related factors, because age may influence the variables under investigation.^26^ Age is a heterogeneous variable that may confound the relationship between UPF intake and health in older adults. Simple adjustments for age may not account for the variability within age groups, potentially leading to biased estimates.^26^ The NutriNet-Santé cohort study associates UPF intake with an increased risk of type 2 diabetes, categorizing participants into age groups of 18-44, 45-59, and ≥60 years.^27^ However, by adjusting for age in the analysis, the study may obscure the negative health effects specific to older adults, because the group aged ≥60 years was not assessed as an individual group.^27^

Although most of the previous research involved observational studies, some randomized controlled trials researched UPFs and health outcomes. A recent systematic review that was focused on randomized controlled trials concluded there were no significant outcomes found in 30 of 42 of the examined health-related outcomes associated with high UPF intakes.^28^

An important feature of healthy aging deserving further investigation is the impact of UPFs on the health of older adults. Given the limited evidence on and the potential advantages and disadvantages of reducing UPF intake, the research evidence from older adults remains ambiguous, particularly considering the contribution of subgroups of UPFs to energy and nutrient intakes.^29–31^ This includes “staple foods” such as breads, cereals, and dairy products, which provide essential nutrients and fiber. Furthermore, studies involving older adults should include relevant outcomes such as disability, frailty, and other geriatric syndromes, because many existing studies that include some older adults do not have these age-specific outcomes. Therefore, the objective of this systematic review was to evaluate the association between UPF consumption and health outcomes among older adults aged ≥60 years.

## METHODS

### Search Strategy and Selection of Studies

Reporting of this systematic review adhered to the Preferred Reporting Items for Systematic Review and Meta-Analyses (PRISMA),^32^ as shown in Table S1. The protocol was registered with the PROSPERO database (identifier CRD42023442783); however, we have made amendments to our PROSPERO-registered protocol to reflect updates based on emerging literature. A systematic literature search was performed in the MEDLINE, Web of Science, Scopus, and Embase databases from inception until June 2023. The search strategy included the following keywords: (“ultra processed foods*” OR “ultra-processed foods” OR (“ultra processed foods*” OR “ultra-processed foods” OR “Nova classification*” AND (aged OR adult* OR “elderly” OR senior* OR geriatric* OR old* OR pension* OR retire*)). Records were downloaded to Covidence, and duplicates were removed. Covidence is a web-based software platform that simplifies the assembly of a systematic review.^33^ All languages were included, and corresponding authors were contacted to request extra data where applicable.

Two investigators (A.W.W. and F.A.S.) independently assessed articles that were initially eligible. Studies that included a measure of correlation of UPF consumption and any health outcome were included. The title, abstract, and full-text screening of the articles were used to determine eligibility. Table 1 shows the inclusion criteria using the PECOS (population, exposure, comparison, outcome, study design) framework. Studies were considered eligible if they satisfied the inclusion requirements of participants (adults aged ≥60 years), exposure (highest UPF intake), comparators (lowest UPF intake), outcome (any health outcome), and study design (case-control studies, cross-sectional studies, and prospective cohort studies). Studies that included adults and, specifically, older adults and did not undergo a subgroup analysis by age were excluded.

**Table 1.** PECOS criteria for the inclusion and exclusion of studies

Parameter	Criterion
Participants	Observational studies including older adults and excluding children and adults younger than 60 years
Exposure	Studies that assessed the association of high intakes of ultraprocessed foods (using the Nova food classification) specifically by older adults and any health outcome were examined.
Comparators	Compared older adults with lowest ultraprocessed foods intake with those who with the highest UPF intake. Consumption of the most ultraprocessed foods was based on Nova food classification.
Outcome	No specific health-related restrictions will be placed on outcome measures.
Study design	Studies included observational studies such as longitudinal, cross-sectional, and case-control studies. No language or date restriction was included when searching databases. Studies that interpreted results of high ultraprocessed foods as household availabilities instead of intakes were excluded. Studies that included adults and older adults and did not conduct a subgroup analysis by age were excluded.

We contacted 40 authors for data on any subgroup analyses of older adults, because they had published a study that included both adults and older adults but adjusted for age in the analysis. Of these, 15 authors replied but were not able to provide a subgroup analysis of older adults. Review articles, letters to the editor, comments, conference proceedings and articles reporting on randomized controlled trials were also excluded. Disagreements were resolved through consensus with a third investigator (A.F.).

### Data Extraction

Data extraction was carried out by 1 investigator (F.A.S.). Conflicts were resolved through discussion with 2 investigators (A.F. and T.R.H.). The data were extracted from the full-text articles and consisted of the following: first author, year of publication, participants’ age, cohort, country of population, follow-up length in years, assessment of UPF, comparison, exposure variables, health outcome, and main results, including adjustments in statistical analysis.

### Appraisal of Methodological Quality

An appraisal of methodological quality of the included articles was conducted using the Newcastle-Ottawa Scale (NOS) tool for observational studies^34^ and the adapted NOS for cohort studies.^35^ The NOS and adapted NOS consist of 8 questions across 3 domains: selection, comparability, and outcomes, and each question is rated up to 1 star, with the question about comparability rated by up to 2 stars. The NOS score can reach a maximum of 9 stars and the adapted NOS score can reach a maximum of 8 stars. A study with a score <7 was considered of lower quality and studies with a score >7 stars were considered to reflect higher quality, consistent with previous systematic reviews.^17^^,^^36^^,^^37^ Two investigators (F.A.S. and A.F.) independently evaluated the methodological quality of each included study. Disagreements were resolved by consensus, and a third researcher (A.W.W.) was consulted.

### Data Synthesis

A narrative synthesis approach was used to systematically synthesize data within included studies. A meta-analysis was not conducted, because of the minimal number of studies and differing outcome measures. The available evidence was systematically reviewed and interpreted to generate new knowledge and insights on the association of high UPF intake and health outcomes in older adults.

## RESULTS

### Search Results

**Figure 1.**
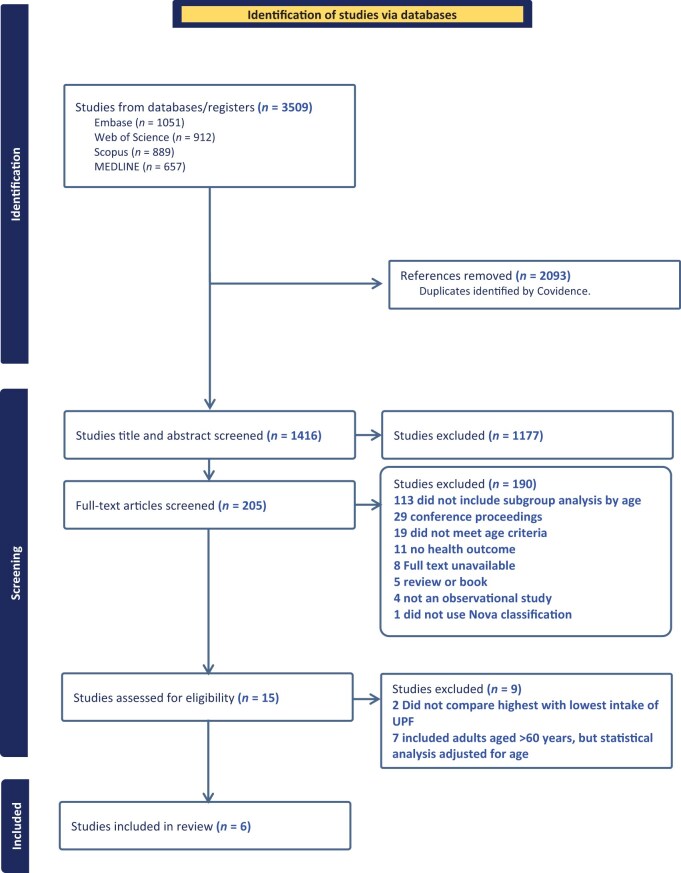
Flowchart of the selection process for the articles included in the systematic review. Abbreviation: UPF, ultraprocessed food.

Figure 1 displays the process of selecting studies according to PRISMA. The search identified 3509 articles, and after excluding duplicates, 1416 studies remained. After abstract and title screening, 205 articles were identified. Of these, 15 studies were assessed for eligibility after full-text screening, and 6 articles were included in the analysis after the selection process.

### Overview and Characteristics of Included Studies

The 6 selected studies were published between 2020 and 2023 and were conducted in 3 countries: the United States (*n* = 1),^38^ Spain (*n* = 4),^39–42^ and Italy (*n* = 1).^30^ Data for all Spanish studies originated from the same participant group using the same cohort. Two of the studies were cross-sectional and observed the association between UPF consumption and nutritional frailty (*n* = 1)^30^ and cognitive performance (*n* = 1).^38^ Four of the studies were of prospective cohorts and examined the relationship between UPFs and the incidence of frailty (*n* = 1),^41^ abdominal obesity (*n* = 1),^42^ renal function decline (*n* = 1),^40^ and incident dyslipidemia (*n* = 1).^39^  Table 2 lists the key characteristics of the included studies.^30^^,^^38–42^

**Table 2.** Characteristics of Included Studies

Reference	Age, y; sample size (sex)	Study design	Follow-up, y	Country (cohort)	Assessment of UPFs	Comparison
Cardoso et al (2022)^38^	≥60 (mean, 69); 2713 (54% female)	Cross-sectional	1	United States (NHANES 2011-2014)	%TE Two 24-h recalls	T3 vs T1
Zupo et al (2023)^30^	≥65 (mean, 73.56); 2185 (49.51% female)	Cross-sectional	8	Italy (Castellana Grotte [Bari, Apulia, southern Italy])	g d^–1^ 2000 kcal^–1^^a^ 85-item self-administered FFQ	Q5 (high) vs Q1 (very low)
Donat-Vargas et al (2021)^39^	≥60 (mean, 68); 1082 (52% female)	Cohort	5-7	Spain (ENRICA)	%TE Computer-based dietary history	T3 vs T1
Sandoval-Insausti et al (2020)^41^	≥60 (mean, 68.7); 1822 (51% female)	Cohort	5-7	Spain (ENRICA)	%TE and g kg^–1^ Computer-based dietary history	Q4 vs Q1
Sandoval-Insausti et al (2020)^42^	≥60 (mean, 67); 652 (44% female)	Cohort	5-7	Spain (ENRICA)	%TE Face-to-face dietary history	T3 vs T1
Rey-Garcia et al (2021)^40^	≥60 (mean 67); 1312 (51% female)	Cohort	5-7	Spain (ENRICA)	%TE and g kg^–1^ d^–1^ Computer-based dietary history	T3 vs T1

Abbreviations: ENRICA, Seniors-Study on Nutrition and Cardiovascular Risk in Spain; FFQ, food frequency questionnaire; NHANES, National Health and Nutrition Examination Survey; Q, quintile; T, tertile; UPF, ultra-processed foods; %TE, percentage of total energy.

a Grams per day of energy intake of 2000 calories per day (standardization was done by dividing the daily food intake into grams per day by calorie intake per day and multiplying it by 2000).^30^

The number of participants ranged from 652 to 2713 across the 6 studies. The mean age of participants was 68.7 years (range, 67-73 years). Two studies had a cross-sectional design and 4 were prospective cohort studies. The duration of the cohort studies was between 3.5 and 8 years.

### UPF Consumption

One of the 2 cross-sectional studies assessed UPF consumption by having participants complete a food frequency questionnaire.^30^ The other cross-sectional study measured UPF intake using two 24-hour recalls.^38^ Three cohort studies used computer-based dietary history,^39–41^ and 1 cohort study used face-to-face dietary history.^42^

The exposure of 1 cross-sectional study was calculated according to grams per day per 2000 calories from UPFs,^30^ and the other cross-sectional study assessed exposure using percentage of total energy (PTE) intake.^38^ Assessment of exposure in 2 cohort studies was through measuring the percent energy of UPF of total energy (TE).^39^^,^^42^ The remaining 3 cohort studies also measured PTE, in addition to determining grams per kilogram of body weight).^40^^,^^41^

Table 3 reports the PTE from UPFs, grams per kilogram of body weight and grams per day consumed by participants as well as health outcomes associated with higher UPF intakes. UPF intakes ranged from an average of 17% to 53% of total intakes measured as PTE or percentage of food weight.^38–42^ One study did not report intakes.^30^

**Table 3.** Health Outcomes Associated With High UPF Intakes

Reference	Study design	Health outcome	UPF consumption	OR (95% CI)	Adjustment	Main results
Zupo et al (2023)^30^	Cross-sectional	Frailty	g d^–1^; mean consumption not reported	1.26 (0.86-1.85), SE = 0.25	Age, sex, education, inflammatory cytokines, alcohol intake, protein to energy ratio, energy intake (standardized) and multimorbidity	Nutritional frailty prevalence was 27%, more frequent in men. ↑ intake of unprocessed or minimally processed foods was inversely related to nutritional frailty. ↑ % of nutritional frailty phenotypes were in high UPF category. Individuals in the high UPF category had a 1.26 times higher probability of nutritional frailty than those in the very low UPF category (OR = 1.26; 95% CI, 0.86-1.85).
Cardoso et al (2022)^38^	Cross-sectional	Mental health (cognitive function)	Mean %TE of UPF was 53%, (range, 32.6%-70.4%) (T1 and T3)	1.37 (−2.72 to −0.03)	Demographics, lifestyle factors, BMI, and depression	↑ UPF intake was inversely associated with worse performance in animal fluency test score (OR = −1.37 [95% CI, −2.72 to −0.03]; *P* .049 for trend) in individuals without preexisting chronic health conditions. No significant associations were observed for those with preexisting chronic health conditions.
Donat-Vargas et al (2021)^39^	Cohort	Incident dyslipidemia	Mean %TE of UPF consumption was 19% ± 11% (T1 and T3)	TG: 1.40 mg/dL (−0.49 to 3.29), *P =* .011 LDL −0.97 (−2.84 to 0.91), *P*-value = .160	Sex, age, total energy intake, educational level (no formal education or primary, secondary, or university), marital status (single, married, widowed/divorced), smoking status (current, former, and never smoker), BMI (<25, ≥25 to 29.9, and ≥ 30), physical activity (inactive, moderately inactive, moderately active, and active), alcohol consumption (nondrinker, former drinker, moderate drinker, and heavy drinker who had an alcohol consumption of ≥40 g d^–1^ in men and ≥24 g d^–1^ in women), fiber intake, number of medications (0, 1-3, >3), and number of chronic conditions (0, 1, ≥2)	↑ UPF intake was associated with more than twice the odds of developing hypertriglyceridemia (OR = 2.66 [95% CI, 1.20-5.90]; *P* .011 for trend), and low HDL cholesterol level (OR = 2.23 [95% CI, 1.22-4.05]; *P* .012 for trend). UPF consumption was not associated with high LDL cholesterol plasma concentration. For each 10% increase in energy intake from UPF, the odds of developing hypertriglyceridemia is 25% higher (OR = 1.25; 95% CI, 0.94-1.66) and 20% higher for low HDL cholesterol (OR = 1.20; 95% CI, 0.97-1.49).
Sandoval-Insausti et al (2020)^41^	Cohort	Incident frailty	%TE of UPF 19.3% (0%-79.7%) (Q1 and Q4)	2.57 g kg^–1^ (1.41-4.70), *P =* .004 %TE: 3.67 (2.00-6.73), *P* < .001	Sex, age (60-69, 70-79, ≥80 y), level of education (primary or less, secondary, university), marital status (single, married, divorced/widower), tobacco consumption (current, former, never smoker), and former-drinker status (yes, no), chronic respiratory disease (yes, no), coronary disease (yes, no), stroke (yes, no), osteoarthritis/arthritis (yes, no), cancer (yes, no), depression requiring treatment (yes, no), and number of medications used (continuous).	UPF energy intake was lower in men (17.7%) than women (20.7%). As UPF increases, frailty risk increases when expressed as %TE (OR = 3.67 [95% CI, 2.00-6.73]; *P* < .001 for trend). As UPF increases, frailty risk increases when expressed as g kg^–1^ (OR = 2.57 [95% CI, 1.41 to 4.70]; *P* .004 for trend).
Sandoval-Insausti et al (2020)^42^	Cohort	Abdominal obesity	Mean %TE of UPF consumption was 17% energy (range, 7%-29%) (T1 and T3)	1.61 (1.01-2.56)	Age, sex, educational level, marital status, smoking, former-drinker status, physical activity in the household and at leisure time, the number of medications consumed per day and the number of chronic diseases diagnosed by a doctor (chronic obstructive pulmonary disease/asthma, coronary heart disease, stroke, heart failure, osteoarthritis or depression), daily intake of fiber, intake of very-long-chain omega-3 fatty acid and the 8-point index of adherence to the Mediterranean diet	↑ UPF intake increases the odds of developing AO by 62% (OR = 1.62 [95% CI, 1.04-2.54]; *P* .037 for trend).
Rey-Garcia et al (2021)^40^	Cohort	Renal function decline	Mean %TE of UPF consumption was 17%. Men: 8.6%-33% Women: 6.8%-29.8% (T1 and T3)	%TE 1.74 (1.14-2.66) 1.62 g kg^–1^ d^–1^ (1.06-2.49)	Sex, age, total energy intake, education level (primary, secondary, university), smoking status (never, former, current smoker), former-drinker status (yes, no), physical activity (MET-h wk^–1^), time spent watching TV (h wk^–1^), and total fiber consumption (g d^–1^), number of chronic conditions (continuous), number of medications used (continuous), hypertension (yes/no), and diabetes (yes/no), hypercholesterolemia (yes/no) and BMI (continuous)	↑ UPF intake increased risk of renal function decline by 74% when expressed as %TE (OR = 1.74 [95% CI, 1.14-2.66]; *P* .026 for trend). ↑ UPF intake increased risk of renal function decline by 62% when expressed as g kg^–1^ d^–1^ (OR = 1.62 [95% CI, 1.06-2.49]; *P* .043 for trend).

Abbreviations: AO, abdominal obesity; BMI. body mass index; LDL, low-density lipoprotein; HDL, high-density lipoprotein; g kg^−1^, gram per kilogram body weight; MET, metabolic equivalent; OR, odds ratio; TG, triglyceride; UPF, ultraprocessed food; %TE, percentage of total energy intake; ↑, increased.

### Association between UPF Intake and Health Outcomes

All 6 studies presented in this review observed a specific adverse health outcome associated with increased UPF consumption. Two studies examined the relationship between UPF consumption and frailty (Table 3).^30^^,^^41^ A cohort study by Sandoval-Insausti et al,^41^ from the Seniors-Study on Nutrition and Cardiovascular Risk in Spain (ENRICA) cohort, focused on incident frailty. The authors found that the highest quartile of PTE of UPF was significantly associated with 3.47 times higher likelihood of experiencing unintentional weight loss, a frailty measure (odds ratio [OR] = 3.47 [95% CI, 1.95-6.17]; *P* < .001 for trend). Associations were found for other frailty components, including little physical activity and slowness (OR = 0.18 [95% CI, 0.14-0.21]; *P* = .048 for trend) and (OR = 0.20 [95% CI, 0.16-0.23]; *P* < .001 for trend), respectively. Similar results were found when measuring UPF in gram per kilogram of body weight, suggesting that increased UPF food intake increases overall risk of frailty.^41^ A cross-sectional study by Zupo et al^30^ had a different method of measuring nutritional frailty, defined in their study as the coexistence of physical frailty as well as a nutritional imbalance. The proportion of older adults with nutritional frailty phenotypes decreased steadily from the highest to the lowest quintiles; the percentage dropped from 21.66% (OR = 1.26; SE = 0.25; 95% CI, 0.86-1.85) to 0.87% (OR = 0.87; SE = 0.15; 95% CI, 0.61-1.22), respectively, indicating that increased consumption of unprocessed or minimally processed foods may protect against nutritional frailty.^30^ When adjusting for confounders such as total protein to energy ratio and alcohol consumption, however, the protective effect of low UPF consumption became less pronounced in nutritional frailty, with decreasing odd ratios across quintiles ([Q1], OR = 0.82; [Q2], OR = 0.82; [Q3], OR = 0.89; [Q4], OR = 1.6; [Q5], OR = 4.06).

In another cross-sectional study that examined the association between UPF consumption and cognition, researchers observed that participants without preexisting chronic health conditions who consumed the highest amount of UPFs had a 1.37-fold increased risk of worse performance in the animal fluency test (a cognitive assessment tool) (OR = −1.37 [95% CI, −2.72 to −0.03]; *P =* .040 for trend).^38^

A cohort study from the ENRICA cohort of 1082 older adults aged 60 years or older found a relationship between high amount of UPF consumption and incident dyslipidemia.^39^ Specifically, participants with the highest UPF consumption had more than twice the odds of developing incident hypertriglyceridemia (≥150 mg dL^−1^; OR = 2.66 [95% CI, 1.20-5.90]; *P =* .011 for trend) or low concentration of high-density lipoprotein (HDL) cholesterol (<40 mg dL^−1^ in men; <50 mg dL^−1^ in women; OR = 2.23 [95% CI, 1.22-4.05]; *P =* .012 for trend) when compared with participants with the lowest consumptions. They also had an increase in TE intake and higher rates of tobacco use compared with those with less UPF consumption No statistical significance was for low-density lipoprotein concentration (>129 mg dL^−1^). Analysis of individual UPF groups did not show any statistical significance between UPF consumption and hypertriglyceridemia or low HDL cholesterol levels.^39^

With regard to renal function decline, a cohort study of 1212 of participants older than 60 years from the same cohort in Spain (ENRICA) measured serum creatinine level to estimate glomerular filtration rate. Renal function decline was defined as an increase in serum creatinine level or a decrease in estimated glomerular filtration rate that was greater than what is expected for age.^40^ After follow-up, the researchers found that participants who consumed the greatest amount of PTE of UPF had a 74% higher risk of developing renal function decline (OR = 1.74 [95% CI, 1.14-2.66]; *P* = .026 for trend) when compared with least amount of UPF consumed.^40^ Statistical significance was found for both PTE from UPF and grams of UPF per kilogram of body weight per day. Although similar results were found when analyzing subgroups based on cardiovascular risk factors (ie, chronic conditions, high blood pressure, diabetes, high cholesterol, obesity), a stronger association was found in individuals with diabetes but without obesity.^40^

Furthermore, another study correlated high UPF intakes with abdominal obesity in the same ENRICA cohort.^42^ Abdominal obesity was calculated based on the World Health Organization criteria of a waist circumference^43^ ≥102 cm and 88 cm in men and women, respectively. Of 652 participants at baseline (years 2008-2010), after a follow-up of 6 years, 177 have developed obesity (years 2012-2015). Participants in the highest tertile of UPF intakes developed a 62% greater association with abdominal obesity (OR = 1.62 [95% CI, 1.04-2.54]; *P =* .037 for trend) when compared with the lowest tertile. Additionally, when comparing participants in the lowest tertile with those in the highest tertile of UPF intake, the latter ingested more TE intake, had lower intake of very-long-chain omega-3 fatty acids, and had a lower adherence to a Mediterranean diet.^42^

### Quality Assessment

Table 4 provides results of assessments of the included articles’ methodological quality according to the NOS.^30^^,^^38^^,^^40–42^ Of the 6 included studies, 4 (also from the same cohort^39–42^) exhibited high methodological quality (NOS score >7), and 2 had a score <7.^30^^,^^38^

**Table 4.** Methodological Quality and Characteristics of Studies Included in the Review

Reference	Selection				Comparability	Outcome			Score
	Representativeness of the exposed cohort	Selection of the nonexposed cohort	Ascertainment of exposure	Demonstration that outcome of interest was not present at start of study	Comparability of cohort based on the design or analysis	Assessment of outcome	Was follow-up long enough for outcomes to occur?	Adequacy of follow-up of cohorts	
Cohort studies									
Sandoval-Insausti et al (2020)^41^	★	★	★	★	★☆	★	★	★	8
Donat-Vargas et al (2021)^39^	★	★	★	★	☆☆	★	★	★	7
Rey-Garcia et al (2021)^40^	★	★	★	★	★☆	★	★	★	8
Sandoval-Insausti et al (2020)^42^	★	★	★	★	★☆	★	★	★	8

Reference	Selection				Comparability	Outcome		Score
	Representativeness of the sample	Sample size	Nonrespondents	Ascertainment of the exposure (risk factor)	The participants in different outcome groups are comparable, based on the study design or analysis.	Assessment of outcome	Statistical analysis	
Cross-sectional studies								
Cardoso et al (2022)^38^	★	☆	★	★	★☆	★	★	6
Zupo et al (2023)^30^	★	☆	★	★	★☆	★	★	6

## DISCUSSION

This systematic review included articles reporting on observational studies that examined the association between UPF consumption (using the Nova classification system) and health outcomes, specifically in studies of older adults aged 60 years or older. Two cross-sectional studies reported a negative association between high UPF consumption on nutritional frailty^30^ and cognitive function.^38^ In terms of cohort studies, high UPF consumption was associated with renal function decline,^40^ incident dyslipidemia,^39^ frailty,^41^ and abdominal obesity.^42^ While these findings suggest a potential relationship between high UPF intake and negative health outcomes, the strength of this evidence is limited because there are a small number of studies published solely in this age group (above 60) and the majority of the studies in this review came from the same cohort (four studies).^39–42^

There may be several reasons why older adults consume UPF, including their lower cost and hyperpalatability’ some of these foods, such as breads and cereals, may have been staples in their diets throughout their lifetime.^44^ Ready-to-consume food products are convenient and affordable, which is the main driver behind their popularity.^44^ This is especially true for older adults, who may be living alone, because they may rely on ready-to-consume, processed meals.^31^ This observation is particularly relevant given that a significant portion of older adults aged >65 years in the European Union reside in a single-person household up to 40.2% of women and 21.8% of men.^45^

All studies in this systematic review showed a negative association between increased UPF consumption and health outcomes. Although there was variability in the specific sources of UPFs across studies in this review, most of the foods consumed in the highest quantile of UPFs were also considered low nutritional-quality foods. This includes energy-dense foods that are high in fat, sugar, and sodium.^46^ For instance, Donat-Vargas et al^39^ found that the highest consumption of UPF came from the following: cookies and pastries (31.2%), processed meat and meat products (15.7%), breakfast cereals and breads (11.1%), and sweets (10.9%). However, no statistical analysis was done to associate those specific food groups with dyslipidemia. Moreover, Rey-García et al^40^ reported that in their study, the highest UPF intakes were from breakfast cereals, nonalcoholic beverages (eg, industrially processed fruit juices), cakes and pastries, and meat products, although no statistical association was found between any of the UPF sub-food groups (eg, breakfast cereals group) and the health outcome of renal function decline in that study. Another study included in this review found that the main food groups contributing to UPF intake were meat and meat products (17.8%), cakes and pastries (12.4%), cookies (11.9%), yogurts and fermented milks (9.5%), jams and confectionary (8.9%), and precooked dishes (7.4%).^41^ Yet, a significant association with incident frailty was found only for the following UPF food groups: yogurts and fermented milks (plain yogurt is not included in this category), cakes and pastries, and nonalcoholic beverages such as instant coffee and cocoa, packaged juices, and other nonalcoholic drinks, excluding soft drinks.^41^

Nonalcoholic beverages also showed a significant linear trend when including tertiles as a continuous variable in the model associated with abdominal obesity.^42^ The UPF food groups that contributed the most to the association of UPF with abdominal obesity were spirits, meat products, and soft drinks, but when comparing those subgroups individually, no statistical significance was found.^42^ Two cross-sectional studies included in this review did not report UPF sources or subgroup associations with health outcomes.^30^^,^^38^

The results of the 6 studies reported in the articles included in this review are consistent with those of a Portuguese study of participants aged ≥65 years, in which the highest UPF contribution came from industrially processed cakes and desserts, yogurt and milk-based drinks, processed meats, and packaged sweet snacks.^47^ Furthermore, a multinational cohort study of participants with a diverse age range (not unique to older adults) concluded that an increase of UPF intake of approximately 260 g d^–1^ (excluding alcohol) was associated with a higher risk of multimorbidity of cancer and cardiometabolic diseases (hazard ratio = 1.09). Interestingly, the association was only found with sugar-sweetened beverages and animal-based UPFs; no association was found with breads and cereals or plant-based alternatives.^48^ This is consistent with recent findings from a study including older women aged ≥60 years in whom high UPF intakes (measured as servings per day) from artificial sweeteners and sugar-sweetened beverages, fat spreads, dairy, and condiments were significantly associated with frailty, but the association was not found in UPF sources coming from whole grains.^49^ Although some UPF sources are rich in free sugars, total fats, and saturated fats, and low in fiber, vitamins, and minerals,^5^ others are rich in fiber and fortified with vitamins and minerals (eg, cereal and cereal products).^14^^,^^23^ These findings challenge the perception that all UPF food sources are detrimental to health and that the impact of UPFs could depend on the specific sub-foods or products within the broad Nova categories, highlighting the need for robust subgroup analyses.^5^^,^^22^^,^^50–53^ Several meta-analyses that assessed observational data sets including adults aged ≥18 years showed that higher UPF intakes were associated with greater risks of abdominal obesity and increased waist circumference.^19^^,^^22^^,^^54^ This is consistent with findings from the present systematic review. Possible explanations for the association could be due to UPFs having low nutritional quality and, therefore, associated with higher energy intakes.^55^^,^^56^^,^^42^

Obesity, alongside tobacco use, insufficient fiber intake, and lack of physical activity, are well-established risk factors for dyslipidemia.^57–59^ This could explain the observed association between high UPF intake and dyslipidemia in this review: participants consuming higher amounts of UPFs also had increase TE intake and higher rates of tobacco use compared with those with lower UPF consumption. Similarly, elevated abdominal fat and body mass index are known risk factors for renal function decline.^60–62^ Our findings in the present review further confirm this association where participants with the highest UPF intakes were associated with around 50% greater risks of renal function decline when compared to those with lowest UPF intakes. This suggests that increased UPF may contribute to increased abdominal fat and body mass index, potentially exacerbating the risk of renal function decline.^63^

In 2 of the 6 studies reviewed in this article, participants who had higher UPF intakes also ate less fiber, fruits, and vegetables than those in lower UPF intake groups.^30^^,^^41^ Negative health effects associated with cognitive function and increased UPF consumption could be due to gut microbiome alteration caused by poor diet quality and inadequate fiber intake, which are associated with diets rich in UPFs, as previously described.^5^^,^^64^^,^^65^ Thus, evidence suggests a possible association between lower diversity and abundance of gut microbiota (gut microbiome depletion) and impaired cognitive function.^66^ This observation might support the reason the included cross-sectional study in this review established a link between high UPF intake and lower performance in cognitive tests—a crucial indicator of cognitive health.^38^ Consistent with the findings of the present review, cognitive function decline was observed in adults aged 35-74 years in a study in which participants in the second, third, and fourth quartiles of daily energy percentage contribution of UPF intake had a 28% accelerated rate of global cognitive decline (β = −0.004 [95%CI, −0.006 to −0.001]; *P* = .003) and 25% accelerated rate of executive function decline (β = −0.003 [95% CI, −0.005 to 0.000]; *P* = .01) compared with individuals in the first quartile.^67^

One of the 6 studies included in this review found a significant association between high UPF consumption and incident frailty, characterized by unintentional weight loss, low physical activity level, and slowness.^41^ Notably, UPF sources with statistical significance in that study were all identified as refined sugar sources, including yogurts and fermented milk (excluding natural, unsweetened options), cakes and pastries, and nonalcoholic beverages such as instant coffee, cocoa, packaged juices, and other nonalcoholic beverages excluding soft drinks. These findings align with those of another study within the same cohort that investigated associations between added sugar intake and frailty in adults aged >60 years.^68^ Researchers on that study observed a greater risk of frailty development in participants with the highest added sugar intake (≥36 g d^−1^) compared with those with the lowest intake (<15 g d^−1^) (OR = 2.27 [95% CI, 1.34-3.90]; *P* = .003 for trend). Furthermore, a dose-dependent increase in frailty components like low level of physical activity and unintentional weight loss was observed with increasing added sugar intake.^68^ This association was particularly strongest for sugars added during food production, which means those foods are also characterized as UPFs. The outcome of unintentional weight loss diverges from research on adults (including both younger and middle-aged populations) for whom intake of UPFs is linked to increased risk of overweight and obesity.^69–73^

A systematic review of data from individuals aged 10-64 years (*n* = 10 studies) provided evidence that high UPF intake significantly increased the risk of being overweight and obese by 2% and 26%, respectively.^71^ Conversely, a cross-sectional study of data on individuals (mean age, 70 years) from the National Health and Nutrition Examination Survey observed that with every 10% increase in UPF energy contribution, there was an increase of prefrailty and frailty risk in both underweight-normal and overweight groups by 2% and 6%, respectively.^74^ However, neither the highest proportion of UPFs by food type consumed nor the exact frailty phenotype was declared in that study. Although poor diet may contribute to increasing risk of frailty, it is important to acknowledge that weakening of muscles and frailty could affect older adults’ physical performance and thereby influence dietary intakes toward more UPF choices for convenience and ease of preparation.^75^ For instance, a study of adults aged ≥65 years highlighted that frailty is linked to lower energy intakes (<21 kcal kg^−1^) and low nutrient intake of protein and vitamins D, C, E, and folate after adjusting for TE.^76^ There is further research evidence that frail adults aged ≥60 years had insufficient dietary intakes when compared with older adults who do not suffer from frailty.^77^ Therefore, older adults living with frailty may be a vulnerable at-risk group for poor nutritional status, and ensuring adequate energy and nutritional intake is imperative for this population group.

An association between high UPF intakes and adverse negative health outcomes, including abdominal obesity, frailty, renal function decline, dyslipidemia, and cognitive function, were identified in this review. However, it is essential to note that UPFs encompass a range of foods that could be favored by older adults, including fortified CCPs. Especially because ultraprocessed CCPs were the most consumed UPF by the very old (aged >85 years) and were the source of the highest amounts of fiber, folate, iron, and selenium.^14^^,^^23^ Additionally, UPF sources in the studies included in this review are mostly from foods of low nutritional quality, and some results differ among cohorts including adults aged ≥18 years (high UPF intake and unintentional weight loss).^30^ Therefore, the conclusions drawn from this review are limited due to the lack of available studies (*n* = 6) to elucidate the specific association between high UPF intakes and health outcomes among older adults. Stronger evidence with a broader range of intakes of UPF sources is important.

### Strengths and Limitations

This systematic review has several strengths. To our knowledge, it is the first systematic review to examine the association between high amounts of UPF consumption and health outcomes specifically in older adults. A robust methodology was followed that adhered to PRISMA guidelines. Also, all included studies had a satisfactory participation rate as well as adequate follow-up rates, where appropriate, which resulted in satisfactory scores when using the risk-of-bias tool for all included studies.

Conversely, there are limitations that should be considered when interpreting the results. First, some of the included studies measured dietary intake using a food frequency questionnaire and self-reported dietary history tools (ie, computer-based dietary history and self-administered food frequency questionnaire), which could initiate dietary recall bias.^78^ These tools can also results in miscalculated UPF intake values because they are not explicitly designed to gather UPF data. In addition, 3 of the 6 studies solely used higher PTE values to quantify UPF intake,^38^^,^^39^^,^^42^ neglecting a grams-per-day measurement. This approach could be susceptible to bias from TE consumption because it could fail to capture true UPF quantity. For example, a participant with a lower overall energy intake might have a higher PTE of UPF intake even if the actual amount of UPF consumed is relatively low. Therefore, measuring UPF as both grams per day and energy per day offers a clearer picture of absolute UPF intake.^79^ Second, the number of studies included are limited (*n* = 6), which reduces the strengths of the findings. Third, in 2 of the 6 included studies, multivariable models were adjusted for TE intakes,^39^^,^^42^ physical activity, and chronic illnesses, which can be a potential limitation, because a sedentary lifestyle can be a habit of those who consume a large amount of unhealthy foods, which also can be associated with negative health status.^80^ Fourth, because this review included data from observational studies, we are unable to provide conclusive evidence directly establishing causality between high UPF intake and health outcomes. Last, 4 of the included studies were from the same cohort (ENRICA),^39–42^ which could indicate biased results because participants share the same characteristics, which limits the application of this reviews findings to a broader population.

## CONCLUSION

On the basis of current knowledge, we have reported the first systematic review, to our knowledge, focused on UPF and health status specifically in adults aged ≥60 years. Higher consumption of UPF was associated with incident dyslipidemia, renal function decline, abdominal obesity, frailty, and poorer cognitive performance in the studies included in this review.

Four of the 6 studies included in this review were from the same cohort, which highlights the lack of diversity in studies in which adults aged ≥60 years were assessed independently of other age groups. In line with the recent UK Scientific Advisory Committee on Nutrition statement on nutrition and older adults, emphasizes the need to evaluate the efficacy of dietary interventions to improve health outcomes for older adults,^13^ further research is required that acknowledges the health effects of UPFs on specific age groups throughout the life course, rather than adults as a homogenous group. This includes properly controlled studies with diet-reporting outcomes designed to assess UPFs, including sensitive subgroup analysis, and physiological, social, and psychological parameters relevant to health outcomes in older adults.

## Supplementary Material

nuae223_Supplementary_Data
