# Supplementary material for: Ultra-Processed Food Intakes and Health Outcomes in Adults Older Than 60 Years: A Systematic Review
Source: Nutr Rev. 2025 Jan 31;83(9):1711–24. doi: 10.1093/nutrit/nuae223 (PMC12343033; doi:10.1093/nutrit/nuae223)
Supplement: nuae223_Supplementary_Data [file nuae223_supplementary_data.doc]

**PRISMA-P (Preferred Reporting Items for Systematic review and Meta-Analysis Protocols) 2015 checklist: recommended items to address in a systematic review protocol***

| Section and topic | Item No | Checklist item |
| --- | --- | --- |
| ADMINISTRATIVE INFORMATION | | |
| Title: |  |  |
| Identification | 1a | Ultra-processed Foods Intake and Health Outcomes in Older Adults: A Systematic Review. |
| Update | 1b | N/A |
| Registration | 2 | This systematic review will be registered in PROSPERO, the international prospective register of systematic reviews. |
| Authors: |  |  |
| Contact | 3a | Title: Prof  Full name: Tom Hill  Position: Prof of Nutrition  Institution: Population Health Sciences  Postal address: Human Nutrition Research Centre, Newcastle University, 2nd floor William Leech Building, Medical School, Framlington Place, Newcastle upon Tyne, NE2 4HH  Telephone number: +44 (0) 191 208 5071  E mail address: [tom.hill@ncl.ac.uk](mailto:tom.hill@ncl.ac.uk)  Title: Dr  Full name: Anthony Watson  Position: Faculty of Medicine  Institution: School of Biomedical, Nutritional and Sports Sciences  Postal address Dame Margaret Barbour Building (Room 5.14). NE2 4DR  Telephone number: (0191) 2089003  E mail address: [anthony.watson@ncl.ac.uk](mailto:anthony.watson@ncl.ac.uk)  Title: Dr  Full name: Andrea Fairley  Position: Lecturer in Human Nutrition and Dietetics  Institution: School of Biomedical, Nutritional and Sports Sciences  Postal address Dame Margaret Barbour Building (Room 5.12). NE2 4DR  Telephone number: +44 (0) 191 208 0298  E mail address: [andrea.fairley@ncl.ac.uk](mailto:andrea.fairley@ncl.ac.uk)  Title Mrs.  Full name Fay Ahmad Shahatah  Position PhD Student  Institution Population Health Sciences  Postal address NE2 4HH  Telephone number +44 7488556493  E mail address f.shahatah2@newcastle.ac.uk |
| Contributions | 3b | Review conception and design: AW, AF, TH, FS  Database searching: FS  Titles and abstract screening: FS, AW, AF (TH will resolve any conflicts)  Full-text screening: FS, AW, AF (TH will resolve any conflicts)  Data extraction: FS will extract data, AW & AF will review extracted data.  Assessment of risk of bias in included studies: FS will appraise ROBINS-I tool, AW and AF will review it.  Drafting the review: FS  Critical revision of the review: AW, AF, TH, FS  All authors will read and approve the final version of the manuscript prior to submission for publication. |
| Amendments | 4 | If we change the protocol, we will modify the PROSPERO registry and describe changes with justifications. |
| Support: |  |  |
| Sources | 5a | University scholarship. |
| Sponsor | 5b | Civil Service Commission, Kuwait Cultural Office UK. |
| Role of sponsor or funder | 5c | The funder has no role whatsoever in developing the protocol. |
| INTRODUCTION | | |
| Rationale | 6 | The NOVA food classification, classifies food into four groups: 1) unprocessed and minimally processed foods, 2) processed culinary ingredients, 3) processed foods and 4) Ultra-processed food (UPF). UPF are foods that are made under several industrial processes, contain little or no wholefoods. Examples of UPF are soft drinks, packaged snacks, ice-cream, breakfast cereals and pre-packaged meals. (Monteiro et al., 2018). The percentage contribution of UPF to dietary energy intake reached 56.8% in the UK population aged 1.5 years or older (Rauber et al., 2018).  Observational studies have shown that high UPF intake is significantly associated with negative health outcomes in adults. Some of the health outcomes include increased risk of obesity, cardiovascular disease, gastrointestinal disorders and metabolic syndrome (Rauber et al., 2021; Srour et al., 2019; Lo et al., 2022; Sandoval-Insausti et al., 2020). Other studies demonstrated that high UPF intake is associated with all-cause mortality in adults and older adults (Rico-Campà et al., 2019; Kim et al., 2019; Blanco-Rojo et al., 2019).  Older adults are consuming less energy than their estimated average requirements (EAR) in the UK (Caireen Roberts and Meadows, 2018). There are several reasons that can cause lower caloric intake such as physical impairment, age-related physiologic changes, diseases and psychological and psychosocial issues (Giezenaar et al., 2016). Lower intakes can eventually lead to malnutrition which can impair quality of life (Eleni Amarantos, 2001, Pirlich and Lochs, 2001). One strategy to enhance nutritional intakes in older adults other than nutrition supplements is food fortification. Most foods consumed by that age group comes from fortified foods which are also reflected as UPF. For instance, UPF sources such as cereal and bread are found to be the highest sources of folate and iron consumed by older adults (Granic et al., 2018). Moreover, yogurts were found to be the highest UPF source in an older adult’s diet in Portugal (Magalhães et al., 2021). Therefore, focusing on this age group can give a better understanding to whether there is sufficient evidence to support the association of UPF intakes in older adults and health outcomes. |
| Objectives | 7 | Understand the influence ultra-processed food intake on older adults in relation to health outcomes. |
| METHODS | | |
| Eligibility criteria | 8 | **PECO:**  **Participants**  Observational studies including older adults. Excluding children and adults below 60 years of age.  **Exposure**  We will examine studies that assess the association of intake of ultra-processed foods (using the NOVA food classification) in older adults and any health outcome.  **Comparators**  Compare older adults exposed to minimally processed foods in relation to older adults exposed to highest intakes of ultra-processed foods based on NOVA food classification.  **Outcome**  No specific health related restrictions will be placed on outcome measures.  **Study Design:**  Studies included will be observational studies such as longitudinal studies, cross-sectional and case-control.  No language or date restriction will be included when searching databases. Non-English studies will be translated by a translator. Studies that interpret results of high ultra-processed foods as household availabilities instead of intakes will be excluded. |
| Information sources | 9 | Scopus, Medline (via PubMed), Embase (via Ovid) and Web of Science. |
| Search strategy | 10 | Databases Scopus, Medline (via PubMed), Embase (via Ovid) and Web of Science will be searched, and an example of search strategy will be included in our PROSPERO registry. Search term will be as follows:  (“ultra processed foods*” OR “ultra-processed foods” OR “ultra processed food*” OR “ultra-processed food” OR “ultraprocessed food” OR “ultraprocessed foods” OR “NOVA classification*” OR “NOVA food classification*” AND (aged OR adult* OR “elderly” OR senior* OR geriatric* OR old* OR pension* OR retire*)).  We will search PROSPERO to avoid conducting similar ongoing or published systematic reviews. Search strategies will be developed by FS with the help of a reviewers experienced in designing and conducting database searches (AW & AF). The entered search terms that are related to ultra-processed foods intakes and health outcomes in those above 60 years of age will be included. |
| Study records: |  |  |
| Data management | 11a | The online review platform, Covidence will be used to manage record and data. Duplicates will be eliminated before screening begins and reviewers will be trained on how to use Covidence. |
| Selection process | 11b | 2-3 reviewers (FS, AW & AF) will work independently to screen titles and abstracts and the entire text according to the inclusion and exclusion criteria. Dissimilarities of opinion will be resolved by discussion and consensus. In case of unresolved disagreements, an additional reviewer will be approached (TH). We will document the reasons if full-text articles were excluded. |
| Data collection process | 11c | The data of identified studies will be independently extracted by one reviewer (FS) and will be validated by another reviewer (AW or AF). Any extra required information will be collected from authors via email. |
| Data items | 12 | Data extraction template will be created using excel and will extract data on the following items:  Authors  Publication year  Journal  Study design  Number of participants  Age (above 60), male/female ratio  Health status of participants  The definition of UPF  Method of assessment of dietary intake  How UPF is measured (g/d or total energy/day)  UPF percentage intake  Follow-up duration  Health outcome  Country |
| Outcomes and prioritization | 13 | Any health outcome, examples include (not limited to):  Obesity, increased waist circumference, increased body fat, hypertension, all-cause mortality, cardiovascular disease, irritable bowel syndrome, Crohn’s disease, depression, frailty, type 2 diabetes, cancer, renal function, metabolic syndrome, liver health, dyslipidemia, cognitive function, multiple sclerosis, dementia, gut microbiota, sleep quality and micronutrient deficiencies. |
| Risk of bias in individual studies | 14 | Risk of bias tool will be Newcastle-Ottawa Scale (NOS) for cohort and case-control studies and an adapted version of the NOS for cross-sectional studies. Risk of bias tool will be measured by FS and reviewed by another reviewer (AW or AF). In case of missing information, the authors will be contacted. If unable to get data from the authors, the risk of bias will be classified as 'unclear'. |
| Data synthesis | 15a | A narrative synthesis of the findings will be done from remained studies after exclusion. Rate of health outcomes data will be described as 95% confidence intervals and odds ratios. Models with the uppermost number of covariates will be chosen if results for more than one model were included in the study. |
|  | 15b | Due to the estimated inconsistency of the design and outcomes assessed across studies, a meta-analysis would not be appropriate. |
|  | 15c | None planned |
| Meta-bias(es) | 16 | The systematic review will comprise a narrative search and not a meta-analysis. |
| Confidence in cumulative evidence | 17 |  |

*** It is strongly recommended that this checklist be read in conjunction with the PRISMA-P Explanation and Elaboration (cite when available) for important clarification on the items. Amendments to a review protocol should be tracked and dated. The copyright for PRISMA-P (including checklist) is held by the PRISMA-P Group and is distributed under a Creative Commons Attribution Licence 4.0.**

*From: Shamseer L, Moher D, Clarke M, Ghersi D, Liberati A, Petticrew M, Shekelle P, Stewart L, PRISMA-P Group. Preferred reporting items for systematic review and meta-analysis protocols (PRISMA-P) 2015: elaboration and explanation. BMJ. 2015 Jan 2;349(jan02 1):g7647.*

References:

CAIREEN ROBERTS, T. S., NATALIE MAPLETHORPE, LORNA COX, SARAH & MEADOWS, S. N., POLLY PAGE, AND GILLIAN SWAN 2018. National Diet and Nutrition Survey results year 7 and 8 (combined) of the Rolling Programme (2014/2015 to 2015/2016). Public Health England.

ELENI AMARANTOS, A. M., AND JOHANNA DWYER 2001. Nutrition and Quality of Life in Older Adults.

GIEZENAAR, C., CHAPMAN, I., LUSCOMBE-MARSH, N., FEINLE-BISSET, C., HOROWITZ, M. & SOENEN, S. 2016. Ageing Is Associated with Decreases in Appetite and Energy Intake--A Meta-Analysis in Healthy Adults. *Nutrients,* 8.

GRANIC, A., MENDONCA, N., HILL, T. R., JAGGER, C., STEVENSON, E. J., MATHERS, J. C. & SAYER, A. A. 2018. Nutrition in the Very Old. *Nutrients,* 10.

MAGALHÃES, V., SEVERO, M., CORREIA, D., TORRES, D., COSTA DE MIRANDA, R., RAUBER, F., LEVY, R., RODRIGUES, S. & LOPES, C. 2021. Associated factors to the consumption of ultra-processed foods and its relation with dietary sources in Portugal. *Journal of Nutritional Science,* 10.

PIRLICH, M. & LOCHS, H. 2001. Nutrition in the elderly. *Best Pract Res Clin Gastroenterol,* 15**,** 869-84.
